# Advances in Functional Scaffolds for Bone and Joint Surgery

**DOI:** 10.3390/jfb16110396

**Published:** 2025-10-22

**Authors:** Ryszard Uklejewski, Mariusz Winiecki, Mikołaj Dąbrowski

**Affiliations:** 1 Department of Constructional Materials and Biomaterials, Faculty of Materials Engineering, Kazimierz Wielki University, 30 Jan Karol Chodkiewicz Street, 85-064 Bydgoszcz, Poland; 2 Adult Spine Orthopaedics Department, W. Dega Orthopaedic and Rehabilitation Clinical Hospital, Poznan University of Medical Sciences, 135/147 28 Czerwca 1956 Street, 61-545 Poznan, Poland; mdabrowski@ump.edu.pl

## 1. Introduction

One of the ultimate goals of bone and joint surgery is reconstruction via implantation of a device that replaces bone and/or joints affected by disease, traumatic damage, or deformity. Reconstruction of large osseous defects—which remains a significant clinical challenge—is being addressed through the development of 3D scaffolds: designed architectures that promote native tissue regeneration and serve as templates for bone tissue engineering [1,2,3]. The functional scaffolds for bone and joint repair are biomaterial constructs constituting advanced implants designed to provide structural support and mechanical stability to a defect site while simultaneously stimulating tissue regeneration and integration. Recent advances in biomaterials science, engineering, manufacturing, and design strategies allow us to tackle this challenge by creating functionally graded scaffolds that mimic bone anatomy, with tailored pore-size distributions and gradual structural and mechanical properties across different morphological regions of a joint to facilitate load bearing [4,5,6,7,8,9]. Beyond structurally functional (osteoconductive) designs, ongoing efforts aim at developing osteoinductive scaffolds with enhanced interactions with host tissues [10,11]. Such functional (or multifunctional) scaffolds are often seeded with mesenchymal stromal/stem cells [12,13,14,15] and combined with biomolecules or growth factors that provide biological signals to stimulate the proliferation and osteogenic differentiation of bone-marrow-derived mesenchymal stem cells [16,17,18,19,20] and osteoblast-like cells, thereby promoting bone regeneration [21,22]. At the same time, such scaffolds are being engineered to inhibit local bacterial infection (either intrinsically antimicrobial or loaded with antibiotics, antimicrobial peptides, metallic ions, or nanoparticles) [23,24,25] and, when appropriate, to support adjuvant bone cancer therapies (preventing tumor recurrence) [26] through approaches such as magnetic hyperthermia [27], photothermal therapy [28], and/or localized drug delivery [29]. Key characteristics of functional scaffolds for bone and joint repair include a porous, interconnected structure for cell and nutrient transport, appropriate biodegradability matching the rate of new skeletal tissue formation, and tailored mechanical properties, such as strength and elasticity, suitable for the specific defect being repaired.

This Special Issue aims at presenting and discussing the latest advancements in functional scaffolds for bone and joint surgery, and includes contributions on topics including, but not limited to, (1) methods for functionalizing scaffolds to support a variety of in vivo functions, (2) the development of functional 3D scaffolds within tissue engineering, (3) novel biomaterials and biofactors for functional scaffolds, (4) conventional and advanced technologies for functional 3D scaffold engineering, and (5) applications of functional scaffolds in surgical treatment procedures.

The Special Issue ‘Functional Scaffolds for Bone and Joint Surgery’ presents six scientific contributions submitted by scholars with renowned backgrounds in scaffold design, fabrication, functionalization, or clinical applications [30,31,32,33,34,35,36,37,38,39,40,41]; it includes one review and five original research articles.

## 2. Special Issue Highlights

Todd et al.’s [42] comprehensive review provides a timely and authoritative synthesis that aligns closely with the aims of this Special Issue. The authors systematically examine the state of the art in functional scaffold design, encompassing material selection (ceramics, polymers, and composites), macro- and microarchitectural optimization, and advanced fabrication strategies such as additive manufacturing and emerging bioprinting techniques. Particular attention is paid to surface functionalization, the controlled incorporation of bioactive molecules and ions, and post-fabrication treatments that modulate cell–material interactions. Of particular value is the review’s translation-focused perspective: it highlights key bottlenecks—such as the trade-off between mechanical competence and porosity, the lack of standardized preclinical metrics, and the challenges of scalable manufacturing—while outlining pragmatic routes to clinical implementation, including multifunctionalization for infection control and local therapy. By integrating evidence from in vitro, in vivo, and early clinical studies, Todd et al. distill both short-term priorities (robust bioactive delivery and reproducible architectures) and long-term directions (patient-specific, graded scaffolds with integrated sensing and therapeutic functions). Overall, the review serves not only as a rigorous primer but also as a strategic roadmap, situating novel experimental reports and clinical case studies within the broader trajectory of regenerative orthopedics.

Pudełko-Prażuch et al. [43] report on the design and fabrication of three-dimensional (3D) porous polymer scaffolds functionalized with β-tricalcium phosphate (TCP) particles using a gel-casting approach combined with rapid heating. The authors prepared five distinct polymer-blend compositions based on poly(lactic acid) (PLA) with embedded TCP and tailored with either poly(ethylene glycol) (PEG, Mn = 600 or 2000 Da) or poly(ε-caprolactone) (PCL) to tune degradation behavior. The resulting porous composite scaffolds exhibited open porosity in the range of ≈58–65% and pore sizes spanning roughly 100–1500 µm (median < 300 µm). Physicochemical characterization and microscopy confirmed homogeneous TCP distribution and a hierarchical microstructure, and in vitro assays showed that scaffold extracts were non-cytotoxic to L929 fibroblasts (indirect/extract test) while direct contact cultures supported MG-63 adhesion and metabolic activity, leading to the identification of the most promising compositions for bone tissue-engineering applications in terms of microstructure, cytocompatibility, and susceptibility to degradation. The authors conclude that gel-casting combined with a rapid heating route is effective for producing highly porous, bioactive polymer–ceramic scaffolds whose degradation rate and surface properties can be modulated by polymer blending to meet differing clinical requirements.

With the aim of enhancing the biological performance of scaffolds by improving cell–material interactions, Zumbo et al. [44] propose alginate/hydroxyapatite (Alg/HAp) three-dimensional (3D) porous scaffolds prepared via internal gelation of Alg/HAp solutions followed by freeze-drying; the scaffolds’ surfaces were functionalized through the physical adsorption of fibronectin (FN). The presence of adsorbed FN was verified by a μBCA protein assay and environmental scanning electron microscopy (ESEM), supporting the interpretation that enhanced FN–biomaterial interactions underlie the observed cellular responses. The study demonstrates that FN functionalization positively affects MG-63 osteoblast-like cell behavior: the FN-coated scaffolds showed improved colonization and higher proliferation compared with controls under static culture, and this beneficial effect was even more pronounced under dynamic culture in a perfusion bioreactor, where cells on the FN-functionalized scaffolds adhered more uniformly and exhibited a more spread morphology. Overall, the work indicates that modulation of FN–scaffold interactions is a promising strategy to augment cell colonization and the regenerative potential of Alg/HAp scaffolds; it may therefore support enhanced tissue-regenerative responses, although confirmation in in vivo models is required.

Uklejewski et al. [45] address a key biomimetic consideration in scaffold design: for correct postoperative function and long-term maintenance of endoprosthesis components fixed into surrounding bone by a connecting scaffold, it is crucial—particularly in degenerative disease—to identify the microstructural and mechanical properties of the compromised host bone. The article deals with a prototype titanium-alloy multi-spiked connecting scaffold (MSC-Scaffold) intended for the fixation of components in a new generation of fully cementless hip resurfacing arthroplasties (RAs), presented in monograph [36], and explores how mechanical embedding of this scaffold alters the subchondral trabecular bone of osteoarthritic (OA) femoral heads. Using micro-CT, femoral heads from OA patients were scanned before and after controlled mechanical embedding of the MSC-Scaffold; patients were stratified by BMI into non-obese patient (NOP, BMI < 30) and obese patient (OP, BMI ≥ 30) groups. Morphometric and computed mechanical parameters—bone volume fraction (BV/TV), trabecular thickness (Tb.Th), trabecular number (Tb.N), apparent bone density (*ρ_B_*), compressive strength (*S*), and Young’s modulus (*E*)—exhibited statistically significant changes after embedding, consistent with local bone densification. These changes were deemed favorable for load transfer from the artificial joint surface via the MSC-Scaffold to the periarticular trabecular bone. The authors therefore suggest that the observed increase in local subchondral strength may reduce the risk of implant migration during postoperative limb loading and support further scaffold geometry optimization for safe elastic load transfer.

Sowislok et al. [46] investigated the intraoperative “biologization” of osteoconductive scaffolds by autologous protein adsorption, performing a comprehensive proteomic characterization of β-tricalcium phosphate (β-TCP) and poly(ε-caprolactone)-β-TCP (PCL-TCP) immediately after clinical contact. Samples of both materials were incubated in two clinically relevant ways—directly in the femoral medullary cavity (FC) and indirectly in a surgical tissue collector (BF) harvesting autologous blood, bone fragments, muscle, and fat from ten patients undergoing primary total hip arthroplasty—and surface morphology and protein adsorption were assessed by microscopy and high-resolution Liquid Chromatography–Tandem Mass Spectrometry (LC-MS/MS), followed by bioinformatics. Both materials formed a very large protein corona (>2000 unique proteins), with β-TCP exhibiting higher total protein amounts and PCL-TCP showing greater proteomic diversity and more pronounced, incubation-dependent shifts in protein composition; these differences correlated with surface roughness and wettability. Notably, ex vivo incubation in the tissue collector produced more consistent adsorption patterns and smaller material-specific differences than direct femoral incubation. The study therefore demonstrates that intraoperative exposure to autologous tissues functionally activates bone substitutes via protein adsorption, and that material surface properties together with the clinical incubation environment shape the early biointerface—a manipulable parameter that may be exploited to steer downstream regenerative responses and implant integration.

Beisekenov et al. [47] address the intrinsic design tension in patient-specific scaffolds—reconciling anatomical conformity with structural reliability—by deploying a hybrid, data-driven pipeline for mandibular reconstruction. From CT-derived mandibular geometry, the authors generated a functionally graded Ti-6Al-4V lattice and optimized porosity, screw layout, and strut thickness within a cyber–physical loop that couples high-fidelity finite element modeling (FEM), a millisecond-scale artificial neural network (ANN) surrogate, and a Bayesian network (BN) for uncertainty quantification. Fifteen candidate scaffolds were fabricated by direct metal laser sintering (DMLS) and post-processed by hot isostatic pressing (HIP) for mechanical testing. The results show that FEM provided the ground truth (≈98% prediction accuracy), the ANN reproduced FEM outputs with ≈94% fidelity while evaluating ~10,000 designs in real time, and the BN constrained worst-case failure probability to <3%. Compared with a traditional “FEM-only” workflow, the hybrid pipeline delivered measurable efficiency gains: the selected 55–65% porosity design reduced Ti usage by ≈15%, shortened the design–build–verify cycle by ≈25%, and improved a composite optimization score by ≈20% relative to a solid baseline. Together, these findings demonstrate that integrating physics-based simulation, AI surrogates, and probabilistic risk assessment yields a validated, additively manufactured scaffold that meets surgical timelines and biomechanical targets, providing a transferable blueprint for patient-specific implants.

## 3. Concluding Remarks

The contributions collected in this Special Issue—one comprehensive review and five research articles—summarize recent, practically relevant advances in functional scaffolds for bone and joint repair. Together they (i) present scalable fabrication and functionalization routes to produce tunable, bioactive constructs; (ii) demonstrate strategies for enhancing early cell–material interactions, including intraoperative biologization and ECM-based functionalization; (iii) provide clinically informed evaluation of host bone mechanics and implant embedding; and (iv) introduce data-driven, patient-specific design and manufacturing workflows. Collectively, these works, each tackling distinct aspects of scaffold design and validation, help overcome key translational bottlenecks—such as limited manufacturability, suboptimal biointegration, or insufficient patient-specific validation—and offer concrete, reproducible directions to accelerate clinical translation in bone and joint repair.

Looking ahead, the convergence of data-driven design, intraoperative biologization, and modular multifunctional scaffolds [48,49] delineates a promising direction for the next generation of adaptive, patient-specific functional biomaterial constructs used as advanced implants in various bone and joint surgery treatments. By integrating computational precision with biological responsiveness and surgical practicality, these emerging strategies may ultimately bridge remaining translational barriers and accelerate the clinical application of developed functional scaffolds in bone and joint reconstructive surgery.

## Data Availability

No new data were created or analyzed in this study. Data sharing is not applicable to this article.
